# Paradoxical risk perception and behaviours related to Avian Flu outbreak and education campaign, Laos

**DOI:** 10.1186/1471-2334-10-294

**Published:** 2010-10-12

**Authors:** Hubert Barennes, Aina N Harimanana, Somchay Lorvongseng, Somvay Ongkhammy, Cindy Chu

**Affiliations:** 1 Institut Francophone pour la Medecine Tropicale, Vientiane, Lao PDR; 2 Institut Francophone de Medecine Tropicale, Vientiane, Lao PDR; 3 Health Frontiers, University of Health Science, Vientiane, Lao PDR

## Abstract

**Background:**

In Laos, small backyard poultry systems predominate (90%). The first lethal human cases of highly pathogenic avian influenza (HPAI) occurred in 2007. Few studies have addressed the impact of outbreaks and education campaigns on a smallholder producer system. We evaluated awareness and behaviours related to educational campaigns and the 2007 HPAI outbreaks.

**Methods:**

During a national 2-stage cross-sectional randomised survey we interviewed 1098 households using a pre-tested questionnaire in five provinces representative of the Southern to Northern strata of Laos. We used multivariate analysis (Stata, version 8; Stata Corporation, College Station, TX, USA) to analyse factors affecting recollection of HPAI educational messages, awareness of HPAI, and behaviour change.

**Results:**

Of the 1098 participants, 303 (27.6%) received training on HPAI. The level of awareness was similar to that in 2006. The urban population considered risk to be decreased, yet unsafe behaviours persisted or increased. This contrasted with an increase in awareness and safe behaviour practices in rural areas.

Reported behaviour changes in rural areas included higher rates of cessation of poultry consumption and dead poultry burial when compared to 2006. No participants reported poultry deaths to the authorities. Overall, 70% could recall an educational message but the content and accuracy differed widely depending on training exposure. Washing hands and other hygiene advice, messages given during the HPAI educational campaign, were not recalled. Trained persons were able to recall only one message while untrained participants recalled a broader range of messages. Factors associated with an awareness of a threat of AI in Laos were: having received HPAI training, literacy level, access to TV, recent information, living in rural areas.

**Conclusion:**

We report a paradoxical relationship between unsafe behaviours and risk perception in urban areas, as well as exposure to HPAI training and message misinterpretation. Future educational campaigns need to be tailored to specific target populations and farming styles, for example, small holder farms as compared to commercial farms. Special attention must be given to varying risk perceptions and the risk of misinterpretation of key messages, economic hardship, and real life consequences of reporting.

## Background

Lao People's Democratic Republic (Lao PDR, Laos) is a landlocked, multi-ethnic, multi-lingual, predominantly rural country (73% agricultural) [1]. Like Cambodia, small backyard poultry systems predominate (90%) with a mean of 10-20 birds/household [1,2]. Livestock contributes to 9% of the GDP in Laos [3]. Loss of poultry has a strong microeconomic impact where daily income is 2 USD/day. During the 2004 outbreak, an estimated 69-108 USD/household was lost [3]. The first highly pathogenic avian influenza (HPAI) poultry outbreak in Laos occurred in 2004; the first two human cases (both lethal) in February 2007 [4,5].

In March 2006, in a national survey, we showed a high awareness level of the disease (98%) [6]. Behaviour changed mostly in urban areas and negatively affected consumption, raising, and trade of poultry.

After an HPAI poultry outbreak in July 2006, intensive training was performed throughout the country by WHO, FAO, CARE and UNICEF focusing on four high priority preventive behaviours: a) hand washing, b) cooking, c) reporting, and d) separating poultry species [7].

Few studies have addressed the impact of outbreaks and educational campaigns on a smallholder producer system. Some surveys appear controversial because of excessively high rates of hand-washing and documented reports of dead poultry [8]. We compared public awareness and adoption of preventive behaviours related to the educational campaign and the ongoing outbreak to the 2006 survey.

## Methods

**Figure 1:**
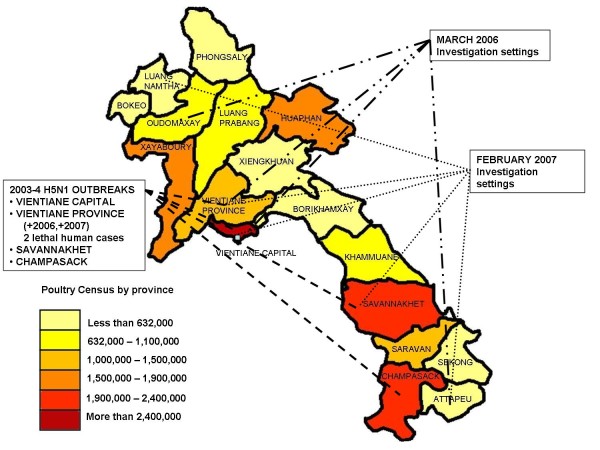
**Map of Lao People's Democratic Republic and 2006-2007 surveys location and previous HPAI outbreaks**.

In February 2007, we conducted a 2-stage household based survey in five provinces representative of the Southern to Northern strata of Laos: Attapeu, Savannakhet Vientiane Capital, Vientiane Province, and Luang Namtha (Figure 1). Urban areas were restricted to 15% of the sample to reflect the rural distribution of the population (Census 2005).

From a list of villages per district, 84 villages accessible by road or motorbike were randomly selected. From a list of households in each village an average of 14 households were selected. One adult > 18 years was randomly chosen from each household. The number of participants was similar in each of the provinces.

Participants were interviewed in Lao language using a standardized questionnaire [6]. We recorded socio-economic characteristics, awareness and knowledge of HPAI, poultry handling, keeping practices, preventive behaviours, and mortality figures [6]. Questions were added regarding the number of poultry currently owned, if training on AI was received, and recollection of key HPAI messages.

Verbal consent was obtained from all participants. The survey was performed with the permission of Lao national, regional and village authorities and in agreement with the Declaration of Helsinski http://www.cirp.org/library/ethics/helsinki/. Ethical approval of from the National Ethical Review Board of Laos is not required for surveys that do not implicate participants. Investigators were doctors from the Institut Francophone de Medecine Tropicale (IFMT) attending a Masters Course with special lectures on Epidemiology, Field Research and Public Health. Pre-tests were performed before conducting the survey.

### Sample size estimation

Based on an estimated 60% perception rate of H5N1 outbreak risk in a 2006 survey in Laos, we used Stata software to calculate the sample size of 988 people with a 5% precision with α = 0.05 and 90% power [6]. To account for incomplete or missing data, an additional 10% of people were included, for a total sample size of 1086 then rounded up to 1100.

### Definitions

We used the 2005 census definitions for urban, semi urban and rural zones: i) urban: People performing no rural activities living in the main cities of Vientiane and Savannakhet; ii) semi-rural: people performing rural activities living near the main cities; iii) rural: people living in the countryside.

### Analysis

Data was entered with Epidata (http://www.epidata.dk, Odense, Denmark) and Stata, version 8 (Stata Cooperation, College Station, TX). First, an univariate analysis was performed using chi-squared and Fisher's exact tests for categorical variables and Student's test for normally distributed continuous data; non parametric tests were used if appropriate. We analysed the factors affecting message recall about HPAI, awareness of HPAI, and behaviour change regarding HPAI according to education, residence and population category, ethnic group, age, family status, sex, occupation, access to TV or radio, presence of the outbreak, and training. All factors with p values ≤ 0.2 were then fit into a multivariable logistic regression model in order to evaluate factors associated with awareness of the threat of AI in Laos and the most important behaviour change reported during the survey (i.e: cessation of poultry consumption.)

We considered P < 0.05 as significant.

## Results

We enrolled 1098 participants (sex ratio F/M: 1.2; mean age: 42.0 years (95% confidence interval: 41-43); illiteracy rate: 7.2% versus 10.0% in urban and rural areas, respectively, p < 0.001). Their socio-characteristics, poultry keeping habits, and HPAI knowledge are shown in Table 1, Table 2, and Table 3, respectively. Less than a third (27.6%, n = 303) had received training on HPAI. Nearly 60% kept poultry at home. Level of immunisation of poultry was low (5.9%) without differences within the zones (Table 2).

**Table 1:** Main characteristics of surveyed population in Laos in 2007

	Urban* 164 (15%)	Semi-U* 364 (33%)	Rural* 570 (52%)	p	Total 1098	95% CI
Sex F	58.5%	57.4%	53.5%	0.3	55.5%	52-58
Age (years)	45.0 +/- 13.5	41.5 +/- 13	41.47 +/- 13	0.008	42.0 +/- 13.5	41-43
Illiterate	7.3%	10.9%	10%	< 0.000	10.2%	8-11.9
Occupation^μ^						
-Housewife	28.6%	8.5%	10%	< 0.000	12.3%	10.4-14
-Trader	17.0%	15.0%	15.9%	> 0.5	15.6%	13.5-17.9
-Farmer	0	43.6%	39.6%	< 0.000	35.0%	32-37
-Civil Servant	12.2%	10.9%	12.1%	0.8	11.7%	9.9-13
-Worker	12.8%	6.8%	5.7%	0.2	7.3	5.9-9.0
-None	5.4%	3.5%	5.1%	0.48	4.6%	3-6

*Defined according to Lao Census 2005: i)Urban: People living in the main cities of Vientiane and Savannakhet and performed no rural activities, ii)Semi U: semi rural people living near the main cities with rural activities, iii)rural people living in the countryside. ^μ^Main occupations

95% CI: 95% Confidence Interval

**Table 2:** Poultry practices in Laos in 2007

	Urban* 164 (15%)	Semi-U* 364 (33%)	Rural* 570 (52%)	*p*	Total 1098	95% CI
Keep Poultry	50.6%	55.4%	64%	0.002	59.2%	56-62
Mean number of hens	5.1 +/- 12.3	5.49 +/- 14.4	6.5 +/- 14.1	0.38	5.9 +/- 13.9	5-7
Mean number of ducks	2.7 +/- 5.3	4.7 +/- 22.1	3.8 +/- 8.1	< 0.000	3.9 +/- 13.	2-3
Poultry deaths (n = 207)						
At least one in the last 2 months**	14%	19.7%	19.6%	0.23	18.8%	16-21
-Mean number of deaths^£^	2.4 +/- 10.4	4.3 +/- 14.5	5.1 +/- 15.8	0.11	4.4 +/- 14.7	4-5
-Estimated loss^μ^	1.6-8	5.6-11.5	7.6-12.9	0.11	3.5-5.3	
Attitudes facing poultry deaths						
-Bury	91.3%	79.1%	91%	0.05	86.9%	81-91
-Throw out	0	1.3%	8%	0.06	4.8%	2-8
-Eat	8.7%	8.3%	2.6%	0.18	5.3%	2-9
-Sell	0	0	0.8%	0.65	0.4%	0.01-2
-Report to authorities	0	0	0		0	
Poultry raising habits (n = 613)						
-Henhouse	1.2%	8.3%	7%	0.09	6.6%	4-8
-Inside the house	3.7%	0.5%	0	0.001	0.6%	0.17-1.6
- < 5 meters from house	71.2%	52.3%	50.2%	0.003	53.6%	49-57
- > 5 meters from house	18.7%	30.8%	33.3%	0.04	30.6%	27-34
No immunization^££^	96.3%	94.7%	92.9%	0.2	94.0	92-95

* Defined according to Lao Census 2005 in method section

^μ^using the compensation value of 18000 kips/hen ≈ 2 US $, the number of poultry deaths/household was 19.3 in 2006.

^£ ^54.1% of poultry keepers experimented poultry deaths in 2006[6]** 18%, 27% and 30% over the last year (0.01)

^££ ^34.2% of poultry keepers reported poultry immunization in 2006[6]

95% CI: 95% Confidence Interval

**Table 3:** Bird flu knowledge in Laos in 2007

	Urban* 164 (15%)	Semi-U* 364 (33%)	Rural* 570 (52%)	*p*	Total 1098	95% CI
Bird flu knowledge						
Never heard of bird flu	4.8%	5.4%	9.4%	0.03	7.4%	5.9-9
Heard from						
-TV	74.3%	81.8%	74.9%	0.03	77.1%	74-79
-Radio	13.4%	13.7%	27.9%	< 0.000	19.6%	17-22
-Paper	5.4%	2.4%	3.3%	0.20	0.206%	0.2-0.4

Avian Influenza risk perception						
-in Laos	49.3%	58.7%	60.3%	0.04	58.2%	55-61
-at home	60.3%	65.1%	68.2%	0.15	66%	63-68
Think human disease risk is higher than 2006	22.5%	43.1%	39.1%	< 0.000	37.9%	35-40

Able to describe at least one sign of AI in poultry	42.68%	61.85%	44.56%	< 0.000	50.00%	47-52

Main reason for higher risk perception n = 383						
- Outbreak in Laos	48.4%	27.8%	42.8%	0.007	37.6%	32-42
- smuggling/importation	9%	14.9%	15.2%	0.64	14.6%	11-18
- lethal disease in human	7.9%	16.2%	14.5%	0.03	14.1%	12-16
- no disease control	3%	9.5%	4.4%	0.11	6.2%	4-9
- seasonal	9%	18.1%	1.4%	0.007	4.7%	2-7
- outbreak/other countries	5.4%	3%	4.7%	0.83	4.4%	3-7
- human deaths	0	2%	7.3%	0.03	4.7%	2-7
- no animal control	6%	7.4%	1.9%	0.03	4.4%	2-7

* Defined according to Lao Census 2005 in method section

95% CI: 95% Confidence Interval

In 2006, 89%-93% of survey participants had heard of avian influenza. In 2007, the level of awareness was similar; 91%-95% (Table 3). The urban population had a decreased risk perception. In rural areas television and radio were the primary communication means. Other communication means such as medical staff, leaflets or posters were rarely reported.

417 (37.9%) believed the risk of HPAI in Laos was higher than in 2006. This was related to the presence of an HPAI outbreak (37.6%), and rarely to the risk of death (< 5%).

Compared to 2006, participants experienced less poultry deaths in the previous 2 months (Table 2). Reported behaviour changes included higher rates of cessation of poultry consumption and dead poultry burial when compared to 2006 (Table 4 and 2). No participants reported poultry deaths to the authorities.

**Table 4:** Behaviour change regarding bird flu in Laos in 2007

	Urban* 164 (15%)	Semi-U* 364 (33%)	Rural* 570 (52%)	*p*	Total 1098	95% CI
Changed behaviour	12.5%	37.1%	50.2%	< 0.000	62.4%	59-65
-Stop eating chicken	35.9%	37.0%	40.5%	0.42	38.7%	35-41
-Avoid contact	3.6%	12.9%	8.4%	0.002	9.2%	7-11
-Stop keeping poultry	3.0%	6.3%	5.4/%	0.30	5.3%	4-6.8
-Wear mask	0.6%	0.2%	1.7%	0.07	1.0%	0.5-1.9
-Clean hands after contact	1.2%	3.5%	2.2%	0.23	2.5%	1.7-3.6
-Eat well cooked chicken	9.1%	28.5%	13.8%	< 0.000	18.0%	15-20

* Defined according to Lao Census 2005 in method section

95% CI: 95% Confidence Interval

Overall 70% could recall an educational message but the content and accuracy differed widely depending on training exposure (Table 5). Trained persons were able to recall only one message while untrained participants recalled a broader range of messages.

**Table 5:** Main messages recalled by the population according to previous training

	Trained^£^ n = 303 (27.6%)	Not trained n = 795 (72.4%)	Total n = 1098 (100%)	OR (IC 95%)
Can recall an educational message	80.2%	66.5%	70.3%	2.03 (1.4-2.8)
Do not eat eggs or sick poultry	33.9%	20.6%	23.5%	2.10 (1.5-2.8)
Must protect oneself when poultry is sick or dead*	6.9%	2.7%	3.9%	2.61 (1.4-4.8)
Dangerous and lethal disease*	18.1%	31.5%	27.6%	0.10 (0.07-0.13)
Disease transmissible to humans*	6.9%	10.8%	9.7%	0.61 (0.3-1)
Outbreak present in Lao PDR	4.6%	10.8%	9.1%	0.39 (0.2-0.7)
Transmission is airborne*	3.9%	8.3%	7.1%	0.45 (0.2-0.8)
There is no immunization available	1.9%	7.1%	5.7%	0.26 (0.1-0.6)
Must cull poultry during outbreak*	3.3%	6.7%	5.8%	0.46 (0.2-0.93)

OR: Odds Ratio 95% CI: 95% Confidence Interval

^£ ^People who had received any formal training on HPAI. The timing and type of training were not elicited. This category represents the reference category for the calculation of the OR.

*Messages included in education campaign

Poultry raising habits did not vary. Poultry immunisation was low (< 6%) in contrast to 2006 (34.2%).

After multivariate analysis, factors associated with an awareness of a threat of AI in Laos and factors affecting behaviour changes are shown in table 6. The presence of HPAI outbreaks did not affect behaviour change. Factors associated with cessation of poultry consumption were: access to TV, having received HPAI training, living in rural areas and age < 45 years.

**Table 6:** Factors associated with H5N1 perception and with changed behaviour*

	**Odds Ratio**^**£**^	***P***	95% Conf. Interval
Believes there is a risk in Laos			

Trained on H5N1	2.5	0.01	1.2-5.2
Literate	2.5	0.000	1.6-4.0
Can recall one message	2.3	0.000	1.7-3.0
Owns a TV	1.9	0.000	1.3-2.7
Heard about H5N1			
in last week	1.5	0.002	1.1-2.0
Live in a rural area	1.4	0.004	1.1-1.9
Male	1.3	0.05	1.0-1.6

Stop eating poultry			

Owns a TV	1.8	0.000	1.3-2.5
Awareness of risk	1.5	0.000	1.2-2.0
Received training	1.4	0.01	1.0-1.8
Farmer	0.6	0.006	0.4-0.8
Age below 45 years	0.6	0.000	0.5-0.9
Keeps poultry	0.4	0.000	0.3-0.5
Lives in a Vientiane Province	0.4	0.000	0.3-0.5

* Multivariate logistic regression

^£ ^The reference category is the category when the presumed risk factor is absent

## Discussion

Our results confirm the trend of awareness and preventive behaviour practice during a time of sporadic human avian influenza infection and after a one year intensive campaign in a small backyard poultry system between 2006-2007. HPAI awareness remained above 90% during this time. In urban areas, risk perception decreased and unsafe behaviours persisted or increased. This contrasted with increased risk perception and decreased unsafe behaviour practice in rural areas, the main target of the national HPAI educational campaign [8].

Preceding our study, poultry outbreaks and human cases (all lethal) initially occurring in urban areas began to occur in semi-urban areas. The presence of outbreaks did not influence the adoption of preventive (safe) behaviours, but did influence risk perception. The unfamiliarity with HPAI in rural areas may also have played a role towards increased risk perception. In contrast, urban areas have become familiar with HPAI and culling controlled previous poultry outbreaks. In rural Thai villages, risk perception was related to familiarity, control of outbreaks, catastrophic potential, and level of knowledge [9]. Telephone surveys performed in Hong Kong showed lower risk perceptions for HPAI in Asia compared to Europe [10]. This was thought to be due to the proximity of the SARS outbreak and/or that the first case of H5N1 did not result in a larger human outbreak [11].

Television nationwide, and radio in rural areas, are a major source of information for HPAI in Laos and Cambodia [2]. High exposure to Thai TV limits the effectiveness of local media messages [8].

Leaflets and posters spread throughout the country and health staff were rarely recognized as a source of information. Posters in Laos often become wallpaper in rural areas thus, their effectiveness in illiterate populations should be evaluated.

Misinterpretation is possible during information campaigns [12,13]. Here, trained people stopped eating poultry more frequently than untrained people. Washing hands and hygiene advices, messages given during the campaign, were not recalled contrasting with another survey among small poultry holders [8].

The estimated losses of poultry in the previous 2 months (Table 2) were lower than estimated losses reported during the 2004 outbreak (69-108 USD) [3]. The economic fragility of the small holder system challenges the feasibility of recommended preventive practices for small backyard farms (estimated cost 75 -100 US$ per household) affecting their survival [12].

Persistence of non reporting is another serious concern [2,6] which was subsequently addressed with systematic compensation during the 2008 outbreaks.

Limitations of this study include recall bias, variability between interviewers, and the possibility that participants did not answer truthfully to sensitive questions.. This study did not address the population living in rural areas not accessible by roads, therefore, rural persons may not be fully represented. Untrained persons were likely to have been exposed to some form of HPAI education or information leading to a contamination effect, which may affect comparisons with trained persons. To decrease variability between interviewers, investigators received specific training prior to survey administration. The questionnaire was pre-tested several times. To decrease recall bias, the period of recalling was restricted to a short duration of time and identified with local feasts and holidays. To decrease the level of false or incomplete responses investigators were not accompanied by Lao authorities allowing confidence between investigators and interviewees to be established. Prior to informed consent, interviewees were free to choose whether or not to participate. After informed consent was obtained, they were made aware that their responses would be confidential.

## Conclusion

Risk perception and adoption of preventive behaviours are motivated by different factors. Controlling outbreaks, addressing misconceptions, providing education and media campaigns all play a role in the psychology of HPAI. As the prevalence of avian influenza outbreaks increase, familiarity increases and the ability to control outbreaks improves. Future educational campaigns need to be tailored to specific target populations and farming styles such as small holder farms versus commercial farms. Special attention must be given to varying risk perceptions, misinterpretation of key messages, economic hardship, and real life consequences of reporting.

## Competing interests

The authors declare that they have no competing interests.

## Authors' contributions

HB wrote the manuscript and is responsible for the overall coordination, design and analysis of the survey. He is also guarantor. AH, SL, SO participated in the study design, collected and analysed the data. CC participated in the analysis and revised the manuscript. All authors contributed to the writing of the paper and read the final version.

## Pre-publication history

The pre-publication history for this paper can be accessed here:

http://www.biomedcentral.com/1471-2334/10/294/prepub
